# Optical Absorption and Scattering Properties at 900–1650 nm and Their Relationships with Soluble Solid Content and Soluble Sugars in Apple Flesh during Storage

**DOI:** 10.3390/foods9121881

**Published:** 2020-12-17

**Authors:** Li Fang, Kangli Wei, Li Feng, Kang Tu, Jing Peng, Jiahong Wang, Leiqing Pan

**Affiliations:** 1 College of Food Science and Technology, Nanjing Agricultural University, Nanjing 210095, China; 2019108040@njau.edu.cn (L.F.); 2016108043@njau.edu.cn (K.W.); fengli@njau.edu.cn (L.F.); Kangtu@njau.edu.cn (K.T.); jpeng@njau.edu.cn (J.P.); 2 College of Light Industry and Food Engineering, Nanjing Forestry University, Nanjing 210037, China; njfuwjh@126.com

**Keywords:** apple flesh, absorption, scattering, soluble sugars, 905–1650 nm

## Abstract

Soluble solid content (SSC) is regarded as the most significant internal quality associated with the taste and maturity in fruits. Evaluating the relationship between the optical properties and soluble sugars facilitates exploration of the mechanism of optical techniques in SSC assessment. In this research, absorption coefficient (*μ_a_*) and reduced scattering coefficient (*μ*′*_s_*) of Fuji apple during storage were obtained using automatic integrating sphere (AIS) at 905–1650 nm. Relationships between *μ_a_*, *μ*′*_s_* and SSC, and soluble sugars contents, were investigated. The result showed that SSC, the content of total soluble sugars (TSS), fructose, glucose and sucrose were all decreasing after storage, and the same trend appeared in the change of *μ_a_* and *μ*′*_s_*. In the whole wavelength range, both *μ_a_* and *μ*′*_s_* were positively related to SSC and soluble sugars contents. The correlations between *μ_a_* and SSC, and soluble sugars contents, showed increasing tendencies with increasing wavelengths, while for *μ*′*_s_*, correlations had the opposite trend. The strongest correlations between *μ_a_* and SSC, and soluble sugars contents, were observed in the correlation of *μ_a_* with sucrose, with an *r* of 0.934. Furthermore, a partial least square (PLS) model for sucrose based on *μ_a_* was built with the coefficient of determination of prediction (*R_p_*^2^) and the root mean square error of prediction (RMSEP) of 0.851 and 1.047, respectively. The overall results demonstrate that optical properties at the range of 905–1650 nm could be used to evaluate SSC of apples and this may due to the strong correlation between sucrose content and *μ_a_*.

## 1. Introduction

Apple fruit is highly valued by consumers for its crispy texture and sweet and sour taste. Soluble sugars in apple fruit largely affect sweetness, and they are the main components of soluble solids [1]. Compared with obtaining the content of soluble sugars, it is faster and more economical to measure soluble solids content (SSC). SSC is measured via the fruit juice. Therefore, SSC is used as a significant property for assessing the sweetness of sugars in apples, and the non-destructive evaluation of SSC by optical techniques have been investigated for many years [2,3,4,5,6], inclusive of near-infrared spectroscopy (NIR) and hyperspectral imaging (HSI).

Light transfer in tissues contains absorption or scattering, and the process of interaction mainly depends on the different chemical components and structures of tissues. By collecting light that is reflected back from or transmitted through the tissue based on NIR and HSI techniques, the chemical as well as structural characteristics of tissues can be acquired [7]. The prediction models for SSC can be built based on chemometrics methods by using the captured spectra and the standard measurements, and some satisfactory results have been obtained [8,9,10,11,12]. The calibration models can then be used to predict new samples after establishing and validating. However, it leads to limitation in illustrating the chemical and structural properties of interest from the meaningful information presented in obtained spectra due to the overarching behaviors of absorption and scattering in tissues collected by NIR and HSI techniques which cannot separate these two properties [13]. Besides, the accuracy of NIR and HSI techniques is limited, because the techniques are based on the Lambert-Beer law, which often discards the scattering effect. Furthermore, the obtained properties of reflectance or transmittance are dependent on the instrument types and light source/detecting probe setup [14]. There are, in general, inherent shortcomings with NIR and HSI techniques, which present great challenge in internal quality assessment of agri-food products.

The optical parameters measurement resulted in separate and quantized information of the absorption and scattering properties by calculating the absorption coefficient (*μ_a_*) and reduced scattering coefficient (*μ*′*_s_*) [15,16]. The current measurement techniques are developed to measure absorption and scattering properties, mainly including integrating sphere (IS) [17,18], spatially-resolved (SR) [19], time-resolved (TR) [20] and frequency-domain (FD) [21]. In addition, the techniques are based on the light transfer theory, which involves inverse adding double (IAD), Monte Carlo (MC), and diffuse approximation (DA), and the optical properties can be obtained by IS with IAD [22,23]. Such methods have opened up the opportunity to attribute internal quality parameters, both chemical composition and structure information, to optical properties of tissues for investigating the one to one relationship between them [24,25,26,27]. He et al. [28] applied an automatic integrating sphere (AIS) system combined with IAD strategies to acquiring the *μ_a_* and *μ*′*_s_* spectra of pear tissues in 400–1150 nm. Using a single integrating sphere, Wang et al. [29] estimated the absorption and scattering properties of healthy and diseased onion skin and flesh over the range of 550–880 nm and 950–1650 nm.

Many studies in Vis-NIR (400–1100 nm) have been conducted. The findings showed that *μ_a_* around 525 and 675 nm were affected by anthocyanin and chlorophyll. In addition, *μ_a_* at 630 nm is attributed to total galacturonic acid (GA) in residue insoluble pectin (RIP), with an *r* of 0.64 [30]. *μ_a_* and *μ*′*_s_* at 675 nm are noticed for area and equivalent diameter of apple tissue cells (*r* = 0.581–0.941) [31]. Nowadays, there are few studies on optical properties in longer wavelength ranges (above 1100 nm). However, the absorption property features above 1100 nm contain more important and obvious bonds, C-H, O-H, C-C, than the features observed in the Vis-NIR range [24,32]. Thus, enhanced sensitivity of optical properties related to tissue components can be obtained compared to the wavelength range above 1100 nm [33,34]. The increase in SSC causes an increase in the signal of absorption at 1198 nm, while water cored tissue caused a characteristic decline in light scattering coefficients [35]. However, the relationship between them has not been quantified, and the relationships between absorption and scattering properties and the compositions in SSC are also not clear yet.

Therefore, this research aimed to comprehensively clarify the mechanism of detecting SSC by optical technology and provide information for accurately developing improved SSC detectors of Fuji apple during storage by optical technology. In the work, the *μ_a_* and *μ*′*_s_* of apple fleshes in longer wavelength range NIR (905–1650 nm) were calculated based on the AIS system with the IAD method, and internal quality (SSC and the contents of soluble sugars) was measured. Finally, relationships between *μ_a_*, *μ*′*_s_* and SSC, the contents of total soluble sugar (TSS), fructose, glucose and sucrose were investigated, then the potential for predicting the SSC was evaluated.

## 2. Materials and Methods

### 2.1. Sample Collection and Storage

The Fuji apples were provided by an orchard in Xuzhou, Jiangsu Province, China. In total, 60 apple samples were stored at 0 ± 1 °C with relative humidity (RH 95%) in normal atmosphere (NA) for 150 days. The measurement took place during 0–150 days, at 30-day intervals and in total 10 individual apples were picked and measured randomly after placing at room temperature for 5 h on each occasion. Another 60 samples were stored at 25 °C with RH 95% in NA for 50 days, and 10 apples were picked from 0 day at about 10-day intervals up to 50 days.

### 2.2. Optical Properties

**Figure 1:**
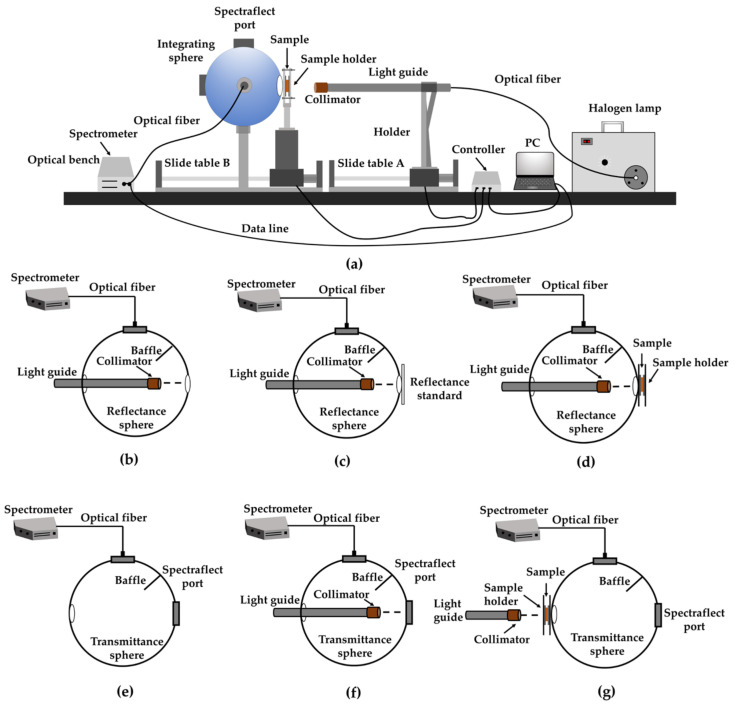
Illustration of the setup of automatic single integrating sphere (**a**), and the schematics for measurements on the reflectance of dark noise *R_d_* (**b**), reference *R_r_* (**c**) and sample *R_s_* (**d**), the transmittance of dark noise *T_d_* (**e**), reference *T_r_* (**f**) and sample *T_s_* (**g**).

The automatic integrating sphere system with IAD was employed to measure the *μ_a_* and *μ*′*_s_*. The diffuse reflectance and diffuse transmittance of the samples, reference and dark noise were needed to calculate *μ_a_* and *μ*′*_s_*, and the spectral signals (905–1650 nm) were collected by a spectrometer (SW2520, Isuzu Optics, Taiwan, China). Different measurement configurations were presented in Figure 1, while specific components of instruments, and measurements′ detailed descriptions, are presented by Wei et al. [36] and Prahl [37].

The accuracy of the estimated *μ_a_* and *μ*′*_s_* was validated with both pure water and 1% Intralipid^®^ (Sigma Aldrich, St. Louis, MO, USA). The main ingredients were soybean oil, glycerin and egg yolk phospholipids. In addition, the particle size distribution in the solution was relatively uniform and stable, also very close to the main scattering particles (chylomicrons) in biological tissues. Thus, it was taken as standard scattering resolution. The scattering resolution was established by diluting 20% stock solution with water. The reference *μ_a_* and *μ*′*_s_* values were described by Van et al. [38] and Deng et al. [39]. The performance of this methodology was validated and the results were reported by Wei et al. [36].

According to the Monte Carlo simulation of Ma et al. [35], the penetration depth of light at 1198 nm is 3.3 mm in the whole tissue of Fuji apple. To ensure the penetration effect of light and the sample volume needed for chemical detection, two 2.5 mm thick flesh slices were cut from each sample apple in the opposite side. The samples were then cut into slices with 2.5 mm thickness × 30 mm width × 35 mm length and the thickness of each slice was measured by using digital calipers. The sample slices were then sandwiched with two pieces of quartz glasses to measure the spectral signals. The specific description of measurements can be found in Wei et al. [36] and Ma et al. [40].

### 2.3. SSC and Soluble Sugars Contents Measurements

The juice of the fresh slices was obtained to assess the SSC by a hand-held refractometer (PAL-1, ATAGO, Tokyo, Japan) after spectral measurement. The measurement range, resolution and accuracy of the instrument were 0.0–53.0% °Brix, 0.1% °Brix and ±0.2% °Brix, respectively. In addition, fructose, glucose, and sucrose contents were quantified by high performance liquid chromatography (HPLC) as described further in Wei et al. [36]. For each sample, two grams of apple flesh homogenate was taken in a centrifuge tube with 30 mL of distilled water in a water bath at 80 °C and centrifuged at 12,000 r/min for 20 min. Supernatants were collected and diluted five-fold., and the solution was filtered through a 0.45 μm filter. Sugars were separated by a Zorbax carbohydrate 70-A column (Agilent, Santa Clara, CA, USA) using a solvent of acetonitrile:water (75:25, *v/v*). The flow rate was 0.8 mL/min at 40 °C. Different concentrations of the sugar standard solution were made for glucose, fructose and sucrose, then the content of the three sugars in the samples was established by comparing each peak retention time and peak area with those of the standard.

### 2.4. Statistical Analysis

All the quantitative comparations between the different soluble sugars of the examined apple flesh were subjected to Variance analysis, which were tested for *p* < 0.05 using the SPSS Statistics 20 package (IBM). The correlation between the contents of soluble sugars and soluble solids was investigated using the Pearson method of analysis with significant *p* < 0.05 in IBM SPSS 20. The partial least square regression (PLS) models of SSC, TSS and soluble sugars contents were built based on *μ_a_* and *μ*′*_s_* separately using MATLAB R2010b^®^ in order to choose the most important soluble sugar parameter that determined optical properties of apple flesh. Finally, the model performance was evaluated by the determination coefficient of prediction (*R_p_*^2^) with the root mean square error of prediction (*RMSEP*), and the determination coefficient of calibration (*R_c_*^2^) with the root mean square error of calibration (*RMSEC*).

## 3. Results and Discussion

### 3.1. Validation Results of Optical Properties

**Figure 2:**
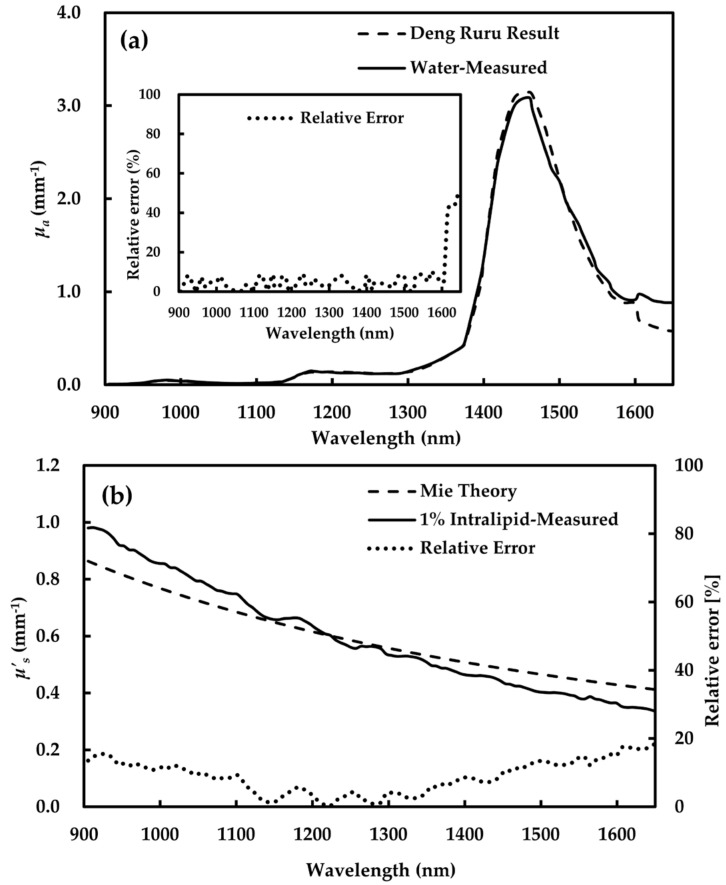
Absorption (*μ_a_*) estimation on water (**a**) and scattering (*μ*′*_s_*) estimation on 1% Intralipid (**b**).

The system in Figure 2a shows the curves of *μ_a_* of water obtained by AIS and *μ_a_* reported by Deng et al. [39]. The results are in agreement with previous reports, which find that there are three absorption peaks at 1485, 1169 and 980 nm. In addition, the relative error of *μ_a_* values at 905–1600 nm ranged from 0.03% to 10.25%. Figure 2b presents the curve of *μ*′*_s_* values of 1% Intralipid based on AIS. A comparison of the measured *μ*′*_s_* and reference value based on Mie theory revealed that both profiles showed a downward trend with the increase in wavelengths [38], and the mean relative error was 9.20%. However, the relative error of *μ_a_* and *μ*′*_s_* after 1600 nm clearly increases. This is due to a low signal-to-noise ratio of the spectrometer, or more detected interference signals. Therefore, *μ_a_* and *μ*′*_s_* at 905–1600 nm were taken as the effective data in this study.

### 3.2. Spectral Analysis

#### 3.2.1. Absorption Coefficient

**Figure 3:**
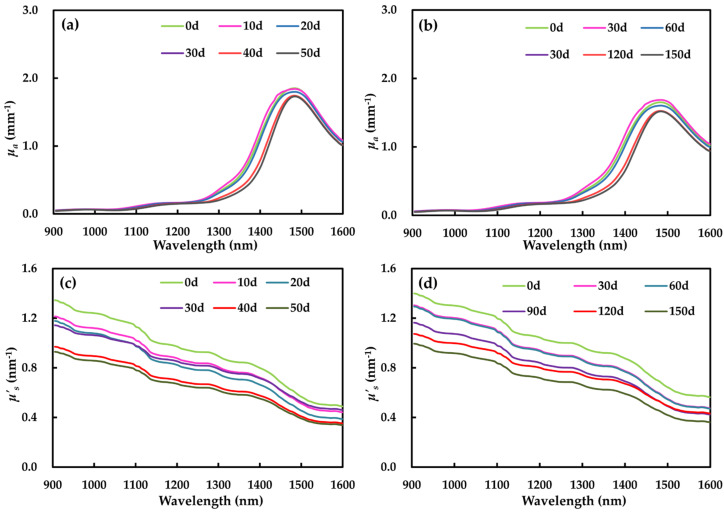
Average optical properties of Fuji apple flesh for six storage times: absorption (*μ_a_*) and scattering (*μ*′*_s_*) at 25 °C (**a**,**c**); absorption (*μ_a_*) and scattering (*μ*′*_s_*) at 0 °C (**b**,**d**). d, day.

The results obtained from the mean *μ_a_* spectra of apple samples storage at 25 and 0 °C are set out in Figure 3a,b. The graphs show that there has been a similar spectral trend, peaking at 980, 1169 and 1485 nm, which corresponds to the vibration absorption of O-H, C-H and C-C [41]. In addition, the absorption at 1485 nm was significantly stronger than that at 980 and 1169 nm, with values (1.52–1.88 mm^−1^) being 10 times higher than that of the others. In addition, the *μ_a_* value peaked at1485 nm when the apple was stored at 25 °C after 10 days. There was no significant difference between day 0 and day 20, and *μ_a_* decreased significantly on the 30th, 40th and 50th days. At 0 °C, *μ_a_* at 1485 nm showed a similar trend with that at 25 °C, with the largest value on the 30th day during storage.

The *μ_a_* values of apple flesh stored at 25 and 0 °C were within 0.04 and 1.88, and within 0.05 and 1.67 mm^−1^, respectively. Generally, the *μ_a_* values in this study were comparable to those reported in other research. In the study by Ma et al. [35], the *μ_a_* values of apple samples were 0.00–0.50 mm^−1^, measured by HIS at 1000–1800 nm. The research of Saeys et al. reported the *μ_a_* values of Royal Gala (0.00–2.20 mm^−1^) based on IS at 350–2200 nm [33]. An important issue that emerged from the data is the dramatic difference in absorption values of apple flesh measured by different techniques. The *μ_a_* obtained using IS is much higher than that with HIS. This may be due to the different sample tissue state, different varieties of apples, different wavelength ranges as well as different measured method in the absorption property [42].

#### 3.2.2. Reduced Scattering Coefficient

Compared with absorption coefficient, the reduced scattering coefficient displays different changing patterns in the spectral region of 905–1600 nm (Figure 3c,d). The *μ*′*_s_* spectral was flatter and steadily decreased with increasing wavelength. In addition, the *μ’_s_* spectral changed consistently as storage time progressed, which was in accordance with studies of He et al. [28] and Cen et al. [31]. The scattering coefficient at 905–1600 nm was in 0.34–1.40 mm^−1^, which was smaller than *μ*′*_s_* of Royal Gala (1.00–1.50 mm^−1^) [33]. This may be related to different apple cultivars [43]. Within the short wave near infrared region (905–1100 nm), the scattering property was still stronger than the absorption property. *μ*′*_s_* was more than seven times the value of *μ_a_*, ranging from 0.80 to 1.40 mm^−1^ at 25 °C, and from 0.04 to 0.11 mm^−1^ at 0 °C. This result was also reported in Wei et al. [36]. In the range of medium wave near infrared (1100–1600 nm), the scattering property gradually declined, and the absorption became the dominant optical property as the wavelength increased [33].

### 3.3. SSC and Soluble Sugars Contents

#### 3.3.1. Changes of Contents of Soluble Solid and Soluble Sugars

**Figure 4:**
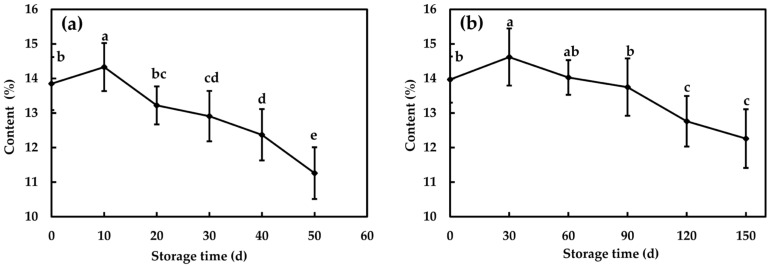
Soluble solid content (SSC) of Fuji apple flesh for six storage times at 25 °C (**a**) and 0 °C (**b**). Data at six storage time with different letters are significantly different with *p* < 0.05. The vertical bars denote standard deviations. d, day.

**Figure 5:**
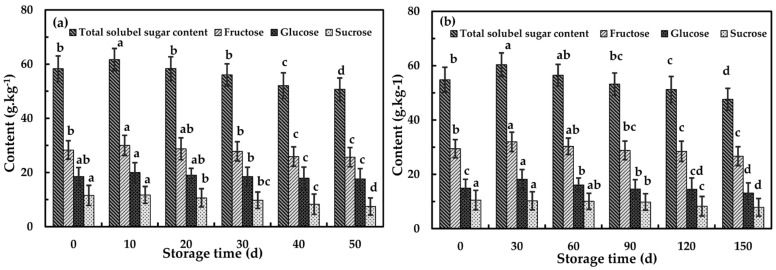
Soluble sugars contents of Fuji apple flesh for six storage times at 25 °C (**a**) and 0 °C (**b**). Columns with different letters for the same cultivar are significantly different with *p* < 0.05. Standard error is indicated. d, day.

At 25 and 0 °C, SSC showed the trend of increasing before decreasing, and separately reached the maximum value on the 10th and 30th day (Figure 4). As shown in Figure 5, the TSS, fructose and glucose contents of flesh increased significantly due to starch hydrolysis, while the sucrose content remained at a high level, with no significant change in the early stage of storage at 25 and 0 °C [44]. This was also consistent with the phenomenon that the fruit will turn sweet during the storage after harvest. After storage, SSC declined significantly (*p* < 0.05), from 13.85% to 11.26% at 25 °C and from 13.97% to 12.26% at 0 °C.

Increasing with storage time, the contents of TSS, fructose, glucose and sucrose gradually declined, and reached the lowest value at the end of storage. At 25 °C, the contents of TSS, fructose and glucose reached the highest value on the 10th day. After 50 days storage, the contents of TSS, fructose and glucose were reduced to 50.72, 25.67 and 17.62 g kg^−1^, respectively. In addition, the content of sucrose decreased from 11.52 to 7.44 g kg^−1^. At 0 °C, the TSS, fructose and glucose contents reached the highest value at a slower rate than at 25 °C, and reached the peak on the 30th day, with levels of 60.34, 31.95 and 18.14 g kg^−1^, respectively. On the 150th day, the TSS, fructose and glucose contents were the lowest, 47.62, 26.66 and 13.09 g kg^−1^, respectively. The content of sucrose did not change significantly during 60-day storage and reached the minimum value of 7.86 g kg^−1^ on the 150th day.

#### 3.3.2. Relationships among SSC and Soluble Sugar Contents

Relationships among the contents of TSS, fructose, glucose, sucrose and SSC were measured on a one to one basis, and SSC presented a positive association profile with TSS, fructose, glucose and sucrose during storage at 25 and 0 °C (*p* < 0.05) in Table 1 and Table 2. It is clear that among TSS and the three soluble sugars, sucrose had the strongest association with the changes of SSC with high corresponding *r* values of 0.973 at 25 °C and 0.963 at 0 °C. Other soluble sugars had lower contributions to the correlations with SSC, shown as a second relation with TSS and fructose, and finally glucose. The results were consistent with the report by Wei et al. [36]. It should be noted that significant positive correlations were found between TSS with three types of soluble sugars (*r* > 0.847), and fructose contributed the most to the correlation with TSS, with *r* values of 0.974 at 25 °C and 0.975 at 0 °C. This could be expected as the content of fructose comprised the largest fraction of the TSS content.

### 3.4. Optical Properties–SSC–Soluble Sugars Contents Relations

#### 3.4.1. Absorption Coefficient–SSC–Soluble Sugars Contents Relations

**Figure 6:**
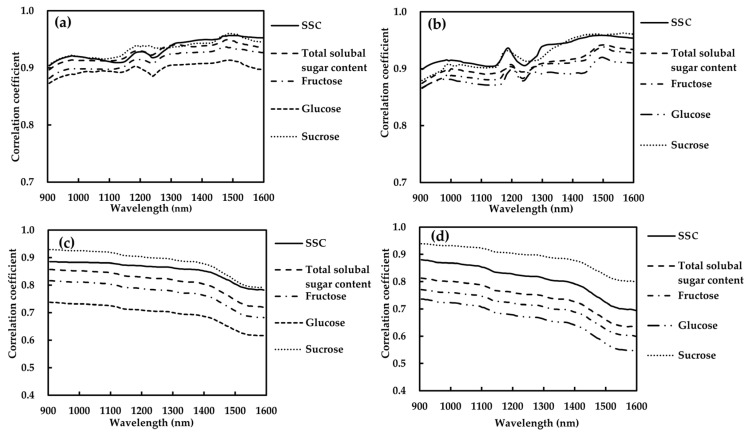
Correlations coefficient of *μ_a_* and *μ*′*_s_* with content of soluble solid (SSC), total soluble sugars (TSS) and three types of soluble sugars of apples at 25 °C (**a**,**c**) and 0 °C (**b**,**d**).

The *r* values between the absorption coefficient of apple flesh at 905–1600 nm and SSC, TSS content and soluble sugars contents at 25 and 0 °C are shown in Figure 6a,b, respectively. The *μ_a_* was positively associated with SSC, TSS content and soluble sugars contents in the whole range, exhibiting high *r* values of 0.873–0.960 at 25 °C and 0.863–0.963 at 0 °C. A previous study reported a similar result, specifically that SSC is positively correlated with *μ_a_* at 1198 nm [35]. At 905–1600 nm, the *r* profile between *μ_a_* and the SSC, TSS content and soluble sugars contents had similar patterns, with almost increasing tendencies with increasing wavelengths. Therefore, from Figure 6a,b, we could observe that SSC and soluble sugars presented stronger correlation in the medium-wave near infrared region than in the short-wave near infrared region. Compared to the report of Wei et al. [36], the average *r* values *μ_a_* and the SSC and TSS content and soluble sugars contents at 905–1050 nm were different. This may be due to different samples used in the two studies and different rates of accumulation or decomposition of soluble sugars. However, due to increasing response to the content information of chemical components, the correlation between absorption properties and soluble sugars increases with the increasing of the wavelength in the whole visible-medium wave near infrared region in general [45].

Overall, SSC (*r* = 0.933) and sucrose (*r* = 0.934) had the highest average correlation coefficients with *μ_a_*, followed by TSS (*r* = 0.928), and finally fructose (*r* = 0.915) and glucose (*r* = 0.899) at 25 ° C. The phenomena may be connected to the strong correlation between SSC and sucrose content. At 0 ° C, *r* values of *μ_a_* and the SSC, TSS content and soluble sugars contents ranged from 0.890 to 0.930. The result also showed that *μ_a_* peaked at 980, 1169 and 1485 nm, and the correlation at the three peaks increased successively, with *r* ranges of 0.881–0.922, 0.894–0.938 and 0.913–0.961, which was in good agreement with the successively stronger spectral signal. To further investigate the correlations with SSC and sucrose content, Table 3 plotted the direct linear correlations of *μ_a_* at 980, 1169 and 1485 nm with SSC and sucrose content (25 °C). The coefficients of determination (*R*^2^) of the equations were between 0.848 and 0.921. Results from the equations indicate that SSC and sucrose content gave good linear correlations with *μ_a_*, with an *R*^2^ of 0.915 and 0.921, respectively, which confirms the results shown in Figure 6a.

#### 3.4.2. Scattering Coefficient–SSC–Soluble Sugars Contents Relations

The values of r between *μ_a_* and either SSC or contents of soluble sugars, when compared with the respective r between *μ*′*_s_* and the same variables, exhibited different patterns; *μ*′*_s_* decreased gradually with the increase in wavelength (Figure 6c,d) which may be related to the decreased values of *μ*′*_s_* over the whole investigated wavelength range. It was also observed that soluble solid and soluble sugars were positively correlated with scattering properties. At 25 °C, the average *r* values of SSC, TSS fructose, glucose and sucrose with *μ*′*_s_* were 0.855, 0.811, 0.771, 0.695 and 0.884, respectively. At 0 °C, the average values of *r* were 0.808, 0.742, 0.704, 0.659 and 0.887, respectively. Moreover, SSC, TSS, fructose and sucrose have higher correlation with *μ*′*_s_* than glucose. Among the three types of soluble sugars, *μ*′*_s_* was most related to sucrose (*r* = 0.789–0.939), followed by fructose (*r* = 0.599–0.816) and glucose (*r* = 0.544–0.738). The same result was found in the correlation of SSC and *μ_a_* with the three soluble sugars. The findings proved that the sucrose contributed the most to the strong relationship between optical properties and SSC.

According to the results of Section 3.4.1 and Section 3.4.2, the correlation between soluble sugars and *μ_a_* was stronger than that between soluble sugars and *μ*′*_s_* in the whole visible-medium wave near infrared region. Besides, with the increase in wavelength, the correlation between soluble sugars and *μ_a_* increased, while the correlation between soluble sugars and *μ*′*_s_* decreased. It could be inferred that *μ_a_* was more associated with chemical components, while *μ*′*_s_* was less correlated with them [7,30,36,46]. Furthermore, previous study presented that scattering property was influenced by microstructure. Rowe et al. [26] measured the optical spectra of apples over the range of 400–1050 nm using IS and obtained an average *r* of–0.68 between the *μ*′*_s_* and firmness. Liu et al. showed that *μ*′*_s_* of kiwifruit had stronger correlation with firmness (*r* = 0.74–0.87) than with SSC (*r* = 0.49–0.81) and moisture content (*r* = 0.59–0.80) [7]. These studies indicated that the absorption and scattering properties had potential to determine the internal quality of apple.

### 3.5. SSC and Soluble Sugars Contents Predictions

Table 4 generalized the statistics of PLS model results of calibration and prediction using *μ_a_* and *μ*′*_s_* at 905–1600 nm for SSC and the contents of soluble sugars of Fuji apple. The *R_c_*^2^ of the modeling set was higher than the *R_p_*^2^ of the prediction set, thus these models were reliable. Among the three prediction models of soluble sugars based on *μ_a_* and *μ*′*_s_*, the best model was for sucrose content (*R_p_*^2^ > 0.7365, *RMSEP* < 1.458 g/kg). In addition, the models relied on *μ_a_*, and the *μ*′*_s_* for fructose and glucose content were slightly worse, the *R_p_*^2^ values of which were in the range of 0.635–0.781 and 0.593–0.725, and the *RMSEP* values were 1.570–2.216 g/kg and 1.344–1.906 g/kg. A previous study reported similar results in that the prediction results on optical properties for sucrose were better than fructose and glucose [36].

In addition, the *R_p_*^2^ values of the five prediction models based on *μ_a_* were in the range of 0.752–0.851, and the *RMSEP* values of the SSC and soluble sugar models were 0.329% and 1.047–2.062 g/kg, respectively. Li et al. [47] assessed the SSC of apple based on near infrared spectroscopy, with the results of *R_p_*^2^ and RMSEP in the ranges of 0.576–0.854 and 0.547–0.673%, and the prediction accuracy of SSC was equivalent to the present study. In addition, according to the model results (Table 4), the prediction results based on *μ_a_* were better than the model results based on *μ*′*_s_*, which further proved that SSC and soluble sugars were more correlated with *μ_a_* than *μ*′*_s_*. The findings obtained from this study made a contribution to the fact the light absorption links to the chemical compositions of materials, and that scattering of photons is a physical procedure that greatly depends on the wave characteristics of photons and the structures of mediums.

In general, the prediction models for sucrose based on *μ_a_* in this study performed well (*R_p_*^2^ = 0.851, *RMSEP* = 1.047). The models for sucrose in 400–1050 nm built with the AIS system, according to the report of Wei et al. [36], were second to the models of our study. According to their study, the models’ *R_p_*^2^ and *RMSEP* values were 0.804 and 1.099, respectively. The result might be caused by the presence of the more obvious bonds C-H, O-H, C-C with a longer wavelength, which indicated that the absorption properties of the mid-wave near infrared region are more suitable for the quantitative detection of soluble solids and soluble sugars.

## 4. Conclusions

The optical properties of Fuji apple during storage in two different storage conditions (25 °C, 50 days; 0 °C, 150 days) were evaluated by the AIS system at 905–1600 nm, and the exact relationships of absorption and scattering properties with SSC and soluble sugars contents were explored. The changes of *μ_a_*, *μ*′*_s_*, SSC and soluble sugars contents showed a similar pattern at the two experimented temperatures during storage. Three prominent absorption peaks at 980, 1169 and 1485 nm were noticed for the spectra of *μ_a_*. In addition, *μ_a_* and *μ*′*_s_* were positively associated with SSC and soluble sugars contents (*r* = 0.659–0.936), and their values are greatly relied on the wavelength. Thus, it could be easily understood that the decreases in SSC and soluble sugar content were accompanied by declines in *μ_a_* and *μ*′*_s_*. Additionally, the *μ_a_* had stronger correlation with these soluble components than with *μ*′*_s_*. Another important conclusion was that both SSC and *μ_a_* were more strongly correlated with sucrose content, with *r* values of 0.933, 0.934 (at 25 °C) and 0.930, 0.927 (at 0 °C). Moreover, partial least square regression (PLS) models for SSC and soluble sugars contents were developed, with the best performance of *R_p_*^2^ = 0.851 and *RMSEP =* 1.047, which appeared in the model of sucrose based on *μ_a_*. Furthermore, due to better measuring bonds of C-H, O-H, C-C at the longer wavelength range, the prediction model at 905–1600 nm performed better than the models developed in the 400–1050 nm range in the report of Wei et al. [36]. Thus, these results made the mechanism for detecting SSC of apples based on optical techniques clear. The strong association between the absorption properties and the sucrose content provided potential and theoretical support in assessing SSC based on optical techniques.

However, this study was carried out on a relatively limited number of fruit and only on apples from one cultivar. Further work needs to involve the investigation of exact relationships between absorption and scattering properties and soluble sugars contents of other varieties of apple flesh, as well as the correlation of optical properties with other components which contribute to quality, such as to firmness.

## Figures and Tables

**Table 1:** Pearson′s linear correlation coefficients among SSC and soluble sugars for 50 days at 25 °C.

	SSC	TSS	Fructose	Glucose	Sucrose
SSC	1	0.956 **	0.921 **	0.868 *	0.973 **
TSS	\	1	0.974 **	0.942 **	0.921 **
Fructose	\	\	1	0.957 **	0.943 **
Glucose	\	\	\	1	0.841 *
Sucrose	\	\	\	\	1

* *p* < 0.05, ** *p* < 0.01, ‘\’: without value, *n* = 60 individual apples; soluble solid content (SSC), total soluble sugars (TSS).

**Table 2:** Pearson′s linear correlation coefficients among SS and soluble sugars for 150 days at 0 °C.

	SSC	TSS	Fructose	Glucose	Sucrose
SSC	1	0.963 **	0.933 **	0.870 *	0.950 **
TSS	\	1	0.975 **	0.967 **	0.847 *
Fructose	\	\	1	0.982 **	0.795
Glucose	\	\	\	1	0.685
Sucrose	\	\	\	\	1

* *p* < 0.05, ** *p* < 0.01, ‘\’: without value, *n* = 60 individual apples.

**Table 3:** Linear fitting equations of soluble solid contents, sucrose contents and the absorption coefficients at 980, 1 189 and 1 498 nm for apple flesh.

Parameters (Independent Variable x)	*μ_a_* (Dependent Variable *y*)	Linear Fitting Equation	*R* ^2^
SSC	980 nm	*y* = 0.0021*x* + 0.0383	0.848
	1189 nm	*y* = 0.0053*x* +0.1039	0.859
	1498 nm	*y =* 0.0581*x* + 1.0107	0.915
Sucrose	980 nm	*y* = 0.0022*x* + 0.0361	0.850
	1189 nm	*y* = 0.005*x* + 0.1064	0.880
	1498 nm	*y* = 0.036*x* + 1.4102	0.921

**Table 4:** Model results of soluble solid contents and soluble sugar contents for apples.

Parameters	Optical Property	Calibration		Prediction	
		*R_c_* ^2^	*RMSEC*	*R_p_* ^2^	*RMSEP*
SSC	*μ_a_*	0.851	0.314	0.833	0.329
	*μ*′*_s_*	0.739	0.693	0.726	0.722
Total soluble sugar	*μ_a_*	0.837	2.055	0.830	2.062
	*μ*′*_s_*	0.710	3.657	0.701	3.699
Fructose	*μ_a_*	0.782	1.567	0.781	1.570
	*μ*′*_s_*	0.658	1.935	0.635	2.216
Glucose	*μ_a_*	0.769	1.338	0.752	1.344
	*μ*′*_s_*	0.611	1.821	0.593	1.906
Sucrose	*μ_a_*	0.860	1.032	0.851	1.047
	*μ*′*_s_*	0.744	1.437	0.736	1.458

The root mean square error of calibration (*RMSEC*); the coefficient of determination of prediction (*R_p_*^2^); the root mean square error of prediction (RMSEP).
